# Clinical impact and a prognostic marker of early rituximab treatment after rituximab reimbursement in Korean pemphigus patients

**DOI:** 10.3389/fimmu.2022.932909

**Published:** 2022-08-02

**Authors:** Ahreum Song, Jieun Jang, Ayeong Lee, Seo Yeon Min, Sang Gyun Lee, Soo-Chan Kim, Jaeyong Shin, Jong Hoon Kim

**Affiliations:** ^1^ Department of Dermatology, Gangnam Severance Hospital, Cutaneous Biology Research Institute, Yonsei University College of Medicine, Seoul, South Korea; ^2^ Hinda and Arthur Marcus Institute for Aging Research, Hebrew SeniorLife, Harvard Medical School, Boston, MA, United States; ^3^ Department of Hospital Administration, Yonsei University Graduate School of Public Health, Seoul, South Korea; ^4^ Department of Dermatology, Yongin Severance Hospital, Cutaneous Biology Research Institute, Yonsei University College of Medicine, Seoul, South Korea; ^5^ Department of Preventive Medicine and Institute of Health Services Research, Yonsei University College of Medicine, Seoul, South Korea

**Keywords:** pemphigus, rituximab, reimbursement, biomarker, autoimmune bullous diseases

## Abstract

Pemphigus is an autoimmune mucocutaneous blistering disease caused by autoantibodies against desmogleins. Rituximab effectively treats pemphigus by inducing remission and rapidly reducing corticosteroid dosage. In Korea, the high cost of rituximab had been a burden until the National Health Insurance began to cover 90% of rituximab costs *via* reimbursement for severe pemphigus patients. We analyzed 214 patients with pemphigus who were treated with their first round of rituximab. The time to initiate rituximab and the time to partial remission under minimal therapy (PRMT) were both significantly shorter after the rituximab reimbursement policy. The total steroid intake for PRMT and complete remission (CR) was less in patients who were diagnosed after the reimbursement. The interrupted time series (ITS) model, a novel analysis method to evaluate the effects of an intervention, showed a decrease in total systemic corticosteroid intake until PRMT after reimbursement began. In peripheral blood mononuclear cells from patients with pemphigus vulgaris, the relative frequencies of desmoglein 3-specific CD11c^+^CD27^−^IgD^−^ atypical memory B cells positively correlated with the periods from disease onset to rituximab treatment and to PRMT and the total systemic corticosteroid intake until PRMT. We found that early rituximab therapy, induced by the reimbursement policy, shortened the disease course and reduced the total corticosteroid use by pemphigus patients. The decreased frequency of circulating desmoglein-specific atypical memory B cells can be used as a surrogate marker for a good prognosis after rituximab.

## Introduction

Pemphigus is an autoimmune blistering disease that affects the skin, the oral cavity, and other mucosal surfaces. Autoantibodies against the cell-surface proteins desmoglein (Dsg) 1 and Dsg3 are pathogenic (1). Intraepidermal blisters with acantholysis and erosion take place, and the failure to manage them can cause life-threatening infections, along with severe pain and poor quality of life (2). Anti-Dsg IgG autoantibodies are necessary and sufficient to induce acantholytic blisters (3, 4). Pathogenic Dsg3-specific B cells have memory phenotypes and undergo somatic hypermutation in pemphigus vulgaris (5), although some germline-reverted antibodies can bind to Dsg3 (6).

The initial treatment of pemphigus involves a high dosage of systemic corticosteroids, which markedly reduces the mortality of patients (7). The combination of high-dose systemic steroids and conventional immunosuppressants, such as mycophenolate mofetil and azathioprine, has been a standard treatment for severe pemphigus for several decades (8). Rituximab is an anti-CD20 monoclonal antibody that depletes B cells and is greatly effective in treating various types of severe pemphigus (9–12). Although rituximab is now used as the first-line treatment for pemphigus in Europe and the U.S (12)., it is still unclear whether early rituximab treatment has a beneficial effect on pemphigus.

Like other biologics, rituximab is an expensive medicine, putting a financial burden on clinicians and patients. Many patients cannot afford rituximab, which causes them to stay on high-dose corticosteroids. In February 2018, South Korea’s National Health Insurance (NHI) began to cover the high cost of rituximab for severe pemphigus patients, in which the government reimburses 90% of the original cost of rituximab. This intervention is referred to as the rituximab reimbursement policy. The study aims to address the clinical effect of early rituximab treatment in pemphigus. We also analyzed the effect of the implementation of the rituximab reimbursement policy on the clinical outcomes of Korean pemphigus patients. Throughout the statistical analysis, a novel model of ITS was used to evaluate the impact of new government reimbursement policies more practically. Furthermore, we examined the peripheral blood mononuclear cell (PBMC) analyses of 17 pemphigus vulgaris patients to identify subtypes of Dsg3-specific B cells related to the prognosis of rituximab treatment.

## Materials and methods

### Study population

For the first part of the study, we collected data on patients who visited the Gangnam Severance Hospital with pemphigus from 1 January 2014 to 31 December 2020 (IRB No. 3-2021-0138). Patients were enlisted if they met the detailed inclusion and exclusion criteria. The inclusion criteria were (i) patients diagnosed as pemphigus vulgaris and pemphigus foliaceus by skin biopsy, and direct and indirect immunofluorescent tests; and (ii) patients who were treated with rituximab under a rheumatoid arthritis protocol (two 1,000-mg biweekly infusions of rituximab). The exclusion criteria included pemphigus patients who did not follow the appointment schedule more than three times after rituximab treatment. In this study, 214 patients were analyzed for time to the first rituximab treatment, total steroid intake over the first year of treatment, total steroid intake over the six months following rituximab treatment, and time from onset to rituximab treatment. To investigate the time to PRMT, total steroid intake to PRMT, and total steroid intake after rituximab treatment until PRMT, we excluded 39 patients who did not reach PRMT after the diagnosis of pemphigus. To measure the total steroid intake to achieve CR, 50 patients were excluded for not reaching CR after the diagnosis. Peripheral blood samples from the patients with 17 pemphigus vulgaris were collected at the start of their first rituximab treatment (IRB No. 3-2019-0191). All the necessary patient information was collected.

### Variables

**Figure 1:**
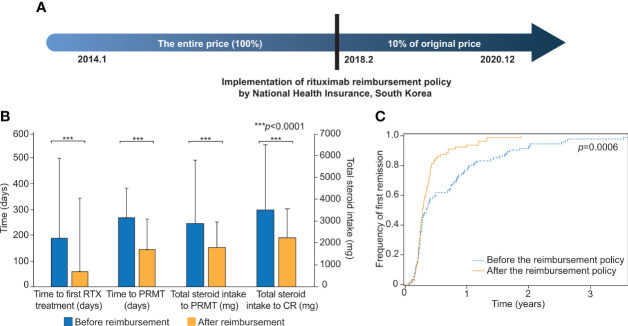
Comparisons of clinical outcomes before and after the implementation of the rituximab reimbursement policy. **(A)** Implementation of the rituximab reimbursement policy by the National Health Insurance of South Korea. Pemphigus patients have paid only 10% of what they used to pay for rituximab treatment since Feb. 2018. **(B)** The comparisons of time to the first rituximab treatment, time to the first PRMT, total steroid intake to PRMT, and total steroid intake to CR are based on the two groups of patients who were diagnosed before and after the implementation of the rituximab reimbursement policy. **(C)** Cumulative incidence curves for the first remission in patients diagnosed before and after the rituximab reimbursement policy. The *p*-value was obtained from Log-rank test. PRMT, Partial remission under minimal therapy; CR, Complete remission.

In February 2018, the NHI of South Korea began to reimburse rituximab for severe pemphigus patients (**Figure 1A**). The dependent variables of this study were time to the first rituximab treatment, total steroid intake over the first year of treatment, total steroid intake over six months following rituximab treatment, time to PRMT, total steroid intake after rituximab treatment until PRMT, time from onset to rituximab treatment, time from onset to PRMT, and total steroid intake to PRMT and CR. The definition of each variable is explained more in detail in **Supplementary Table S1**. PRMT means the moment when the symptoms and general condition of the patient stabilize, so that new lesions are rarely developed and healed within one week under minimal therapy (prednisone ≤10 mg/day). CR indicated the point when the patient maintained a disease-free condition without new lesions under the least amount of prednisone (≤2.5 mg/day).

To evaluate the implementation of rituximab reimbursement for interrupted time series (ITS) analyses, this study included “policy” and “trend after policy” as variables. The “policy” variable was defined as 0 before the implementation of rituximab reimbursement and 1 after the implementation of rituximab reimbursement. This variable refers to a prompt change in rituximab treatment just after the rituximab reimbursement began in February 2018. “Trend after policy” covers the continuous time (in months) starting in February 2018. This refers to the trend in outcomes after the implementation of the policy. For covariates, we adjusted for age (≤29 years, 30 to 39 years, 40 to 49 years, 50 to 59 years, and ≥60 years), sex (male, female), and type of pemphigus (pemphigus vulgaris, pemphigus foliaceus).

### Flow-cytometric analysis

PBMCs were obtained from blood samples of patients using Ficoll–Paque (GE Healthcare, Chicago, Ill). Dead cells were excluded with the LIVE/DEAD Fixable Aqua Dead Cell Stain (Invitrogen, Carlsbad, CA). After blocking with Fc receptor blocking solution (Biolegend, San Diego, CA), we incubated cells with 0.5 µg biotinylated human Dsg3 protein (CUSABIO, Wuhan, China) at 4°C for 1 h and made Dsg3 protein tetramers using streptavidin-BV421 and PE (Invitrogen). Then, the cells were stained with fluorescent-conjugated monoclonal antibody against CD3-BV510 (UCTH1), CD10-AF700 (HI10a), CD11c-BV605 (B-ly6), CD14-BV510 (MφP9), CD19-APC-Cy7 (SJ25C1), CD20-PerCP-Cy5.5 (2H7), CD27-BV711 (M-T271), CD38-PE-Cy7 (HIT2), IgD-BV786 (IA6-2) (all from BD Biosciences, Franklin Lakes, NJ), CD24-PE-CF594 (ML5) (Biolegend), and FcRL5-APC (509F6) (Invitrogen). Cells were detected with a BD LSRFortessa™ X-20 Cell Analyzer (BD Biosciences). For flow cytometric analysis, we have employed an established flow cytometry gating strategy as previously described (13) to detect antigen-specific B cells and atypical memory B cells. The data were analyzed by the FlowJo software package (BD Biosciences), and plots were generated using GraphPad Prism 9 (GraphPad Software Inc., San Diego, CA, USA).

### Statistical analysis

The chi-square test was used to compare demographic characteristics between the two groups. Then, the means and standard deviations of the dependent variables were compared using the t-test. An ITS method using a generalized linear model was applied to investigate the effect of the rituximab reimbursement policy on dependent variables. As previously described in various studies using ITS (14), our regression analysis equation is


$$Y_{it}=\beta 0+\beta 1\times Time_{t}+\beta 2\times Policy_{t}+\beta 3\;\times Time\;after\;policy_{t}+\beta 4\times \;X_{it}+\;e_{it}$$

where *Y* = dependent variables; *i* = each patient; t = time period; *time =* a continuous variable starting in January 2014; *Policy*: a binary variable (0 before the implementation of rituximab reimbursement; 1 after the implementation of rituximab reimbursement); *time after policy*: a continuous variable starting in February 2018; X = independent variables; and e = the error term. The statistical analysis was performed in SAS software, version 9.4 (SAS Institute, Cary, NC, USA). A *p*-value of <0.05 was considered to indicate a statistically significant result. In flow cytometry analysis, the Student’s t-test was used to determine the statistical significance between comparable parameters. Pearson’s correlation analysis was used to measure the strength of relationships between variables. A p-value of <0.05 was considered statistically significant. The statistical analysis of the data was performed using GraphPad Prism 9 (GraphPad Software Inc.).

## Results

### Patient characteristics

A total of 214 patients were included in this study. There were 153 patients with pemphigus vulgaris (71.5%) and 61 with pemphigus foliaceus (28.5%). The male-to-female ratio was nearly 1:1, and the patients who were 50 or older comprised approximately 60% of the whole population. There were 105 pemphigus patients who were diagnosed before the implementation of the rituximab reimbursement policy (49.1%) and 109 patients diagnosed after the implementation (50.9%). There was no statistically significant difference between the two groups in terms of sex, age, year of diagnosis, or type of pemphigus. Patient characteristics are shown in **Table 1**.

**Table 1:** General characteristics of the study population according to the timepoint of diagnosis of pemphigus^1^.

Variables		Total		Before policy (2014.1.–2018. 1.)		After policy (2018.2.–2020. 12.)		*P-*value^2^
Number of patients		214	(100.0)	105	(49.1 of total)	109	(50.9 of total)
**Sex**								0.689
	Male	103	(48.1)	52	(49.5)	51	(46.8)	
	Female	111	(51.9)	53	(50.5)	58	(53.2)	
**Age**								0.944
	≤29	14	(6.5)	8	(7.6)	6	(5.5)	
	30–39	22	(10.3)	11	(10.5)	11	(10.1)	
	40–49	52	(24.3)	26	(24.8)	26	(23.9)	
	50–59	60	(28.0)	30	(28.6)	30	(27.5)	
	60+	66	(30.8)	30	(28.6)	36	(33.0)	
**Year of diagnosis**								
	2014	6	(2.8)	6	(5.7)	0	(0.0)	
	2015	33	(15.4)	33	(31.4)	0	(0.0)	
	2016	36	(16.8)	36	(34.3)	0	(0.0)	
	2017	25	(11.7)	25	(23.8)	0	(0.0)	
	2018	39	(18.2)	5	(4.8)	34	(31.2)	
	2019	44	(20.6)	0	(0.0)	44	(40.4)	
	2020	31	(14.5)	0	(0.0)	31	(28.4)	
**Types**								0.778
	Pemphigus vulgaris	153	(71.5)	76	(72.4)	77	(70.6)	
	Pemphigus foliaceus	61	(28.5)	29	(27.6)	32	(29.4)	

^1^Data shown are number (percentage).

^2^P values are calculated by the chi-squared test.

### Clinical outcomes are improved after the rituximab reimbursement policy

We evaluated the differences in two patient groups: those who were diagnosed before the policy and those who were diagnosed after the policy (**Table 2**; **Figure 1B**). The mean time between the first visit to the clinic and the initiation of RTX treatment was 189 days (SD = 310) before the policy and 59 days (SD = 115) after the policy (*p <*0.0001). The mean value of the total amount of steroids that the patient had taken for the first year of treatment was 2,789 mg (SD = 1,305) before the policy. This was substantially reduced to 2,397 mg (SD = 1,333) after the policy (*p* = 0.031). The total steroid intake over the six months following RTX treatment (1,673 mg vs. 1,467 mg) and the duration from the onset of the disease to the start of RTX treatment (635 days vs. 456 days) also showed lower values in the group after the policy, but neither was statistically significant (*p* = 0.085, *p* = 0.090, respectively).

**Table 2:** Changes in clinical outcomes before and after the implementation of the rituximab reimbursement policy.

Variables	Total	Before policy(2014.1.–2018. 1.)				After policy(2018.2.–2020. 12.)				*P-*value*^1^*
	N	N	Mean	±	SD	N	Mean	±	SD
Time to first RTX treatment (days)	214	105	189	±	310	109	59	±	115	<.0001
Total steroid intake for first year of treatment (mg)	214	105	2,789	±	1,305	109	2,397	±	1,333	0.0309
Total steroid intake for six months following RTX treatment (mg)	214	105	1,673	±	914	109	1,467	±	822	0.0846
Time from onset to RTX treatment (days)	214	105	635	±	822	109	456	±	708	0.0896
Time to PRMT (days)^2^	175	95	268	±	285	80	145	±	118	0.0002
Total steroid intake after RTX treatment until PRMT (mg)^2^	175	95	1,309	±	1,272	80	1,152	±	857	0.3330
Total steroid intake to PRMT (mg)^2^	175	95	2,902	±	2,827	80	1,805	±	1,166	0.0008
Total steroid intake to CR (mg)	164	76	3,573		3,058	88	2,239		1,023	0.0002

^1^P values are calculated by t-test.

^2^Excluding those who did not reach PRMT after diagnosis of pemphigus.

RTX, Rituximab; PRMT, Partial remission under minimal therapy; CR, Complete remission.

Then, the outcomes from both groups were examined, incorporating PRMT. The mean time from the first visit to our clinic to PRMT was 268 days (SD = 285) before the policy, and this was decreased to 145 days (SD = 118) after the reimbursement began (*p =* 0.0002). The mean total corticosteroid intake until the patient reached PRMT was 2,902 mg (SD = 2,827) before the policy and 1,805 mg (SD = 1,166) after the policy (*p =* 0.0008). The mean total corticosteroid intake until the patient achieved CR was 3,573 mg (SD = 3,058) before the reimbursement and 2,239 mg (SD = 1,023) after the reimbursement (*p =* 0.0002) (**Table 2**). The cumulative incidences of the first partial remission for the patients diagnosed before the reimbursement policy and after the reimbursement policy are shown in **Figure 1C**. The time to achieving partial remission was significantly faster among patients diagnosed after the implementation of rituximab reimbursement (*p =* 0.0006 from the log-rank test). There was no significant difference in the percentage of patients who achieved CR, relapsed, and experienced complications between the two groups (**Supplementary Table S2**).

### The rituximab reimbursement policy reduces total steroid intake following rituximab

**Figure 2:**
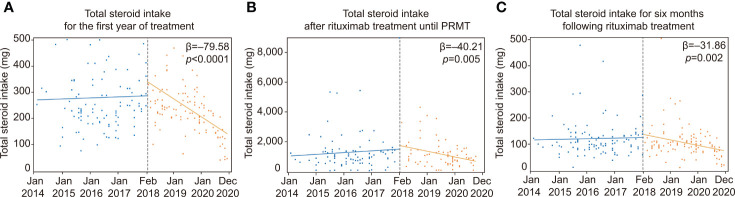
ITS analysis of changes in total steroid intake. ITS analysis of the implementation of rituximab reimbursement. **(A)** Total steroid intake for the first year of treatment before and after the rituximab reimbursement policy. **(B)** Changes in total steroid intake after rituximab treatment until achieving PRMT before and after the rituximab reimbursement policy. **(C)** Total steroid intake over the six months following rituximab treatment before and after the rituximab reimbursement policy. PRMT, Partial remission under minimal therapy; ITS, Interrupted time series.

The implementation of the rituximab reimbursement policy by the NHI triggered a decrease in the total amount of corticosteroid intake throughout the treatment period (**Figure 2**; **Table 3**). The trend in total steroid intake for the first year of treatment was not statistically significant before the policy (*β* = 2.31*, p* = 0.792). The total steroid intake showed decreasing trends after the new reimbursement policy was implemented (*β* = −79.58, *p <*0.0001). There was no significant change in either the level or the trend of total steroid intake until PRMT was reached after the reimbursement policy. Before the policy, the amount of total steroid intake after the administration of rituximab until reaching PRMT and for six months afterward showed a slightly increasing trend, but not a significant one (total steroid intake until PRMT: *β* = 7.11, *p =* 0.380; total steroid intake over six months *β* = 3.38, *p* = 0.594). There was a considerable decreasing trend in the total amount of steroids taken until the PRMT after the introduction of the new policy (*β* = −40.21, *p* = 0.005). The total amount of steroids over the six months after rituximab markedly decreased after the policy (*β* = −31.86, *p* = 0.002). The introduction of the new policy significantly decreased the time from the onset of the disease to the administration of rituximab (*β* = −318.27, *p =* 0.014). However, the time trend after the policy was not significant (*β* = 3.61, *p =* 0.578).

**Table 3:** Results of the interrupted time series analysis for clinical outcomes^1^.

	Time before implementation of RTX reimbursement (by month)			Implementation ofRTX reimbursement				Time after implementation of RTX reimbursement(by month)		
				Before(~2018.01.)	After(2018.02. ~)					
	β	S.E.	*P-*value	β	β	S.E.	*P*-value	β	S.E.	*P*-value
**Total steroid intake over** **first year of treatment (mg)**	2.31	8.76	0.7924	Ref.	1,027.99	330.77	0.0019	−79.58	13.91	<.0001
**Total steroid intake to** **PRMT (mg)^2^**	−15.43	13.67	0.2590	Ref.	382.22	507.41	0.4513	−36.60	22.13	0.0982
**Total steroid intake after RTX treatment until PRMT (mg)^2^**	7.11	8.09	0.3797	Ref.	399.70	333.52	0.2307	−40.21	14.33	0.0050
**Total steroid intake over six months following RTX treatment (mg)**	3.38	6.33	0.5937	Ref.	281.85	224.61	0.2095	−31.86	10.01	0.0015
**Time from onset to** **RTX treatment (days)**	0.92	4.76	0.8466	Ref.	−318.27	128.95	0.0136	3.61	6.49	0.5779

^1^Adjusted factors included sex, age, and type of pemphigus.

^2^Excluding those who did not reach PRMT after diagnosis of pemphigus.

RTX, Rituximab; PRMT, Partial remission under minimal therapy.

### Circulating Dsg3-specific atypical memory B cells are associated with early rituximab treatment and disease prognosis after rituximab treatment

**Figure 3:**
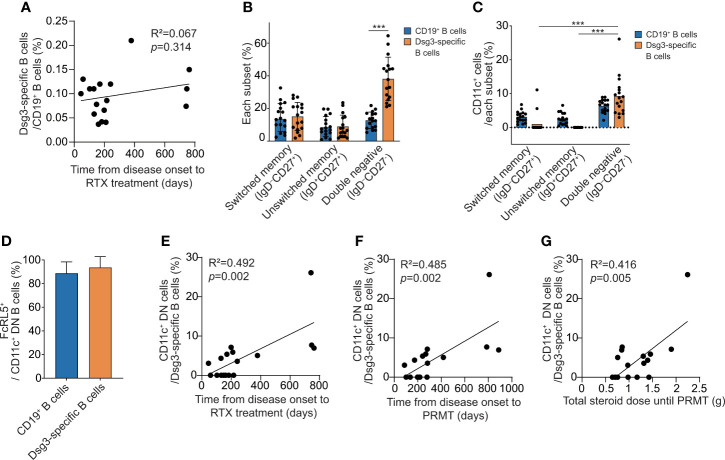
The analysis of Dsg3-specific B cell subsets associated with clinical outcome after rituximab treatment. **(A)** Correlation between the time from disease onset to rituximab treatment and the proportion of Dsg3-specific B cells within CD19^+^ B cells. **(B)** The proportions of switched memory (IgD^-^CD27^+^) B cells, unswitched memory (IgD^+^CD27^+^) B cells and DN (IgD^-^CD27^-^) B cells among CD19^+^ and Dsg3-specific B cells were compared. **(C)** The proportions of CD11c^+^ cells were compared among switched memory, unswitched memory, and DN cells from CD19^+^ and Dsg3-specific B cells. **(D)** The proportions of FcRL5^+^ cells in Dsg3-specific CD11c^+^ DN B cells and CD19^+^CD11c^+^ DN B cells are shown. **(E–G)** Correlation analyses of the proportion of CD11c^+^ cells among Dsg3-specific DN B cells were performed for **(E)** the period from the onset to the administration of rituximab, **(F)** the time from the onset of the disease to reaching PRMT, and **(G)** the total corticosteroid intake until reaching PRMT. ***p <0.0001. RTX, Rituximab; DN, Double-negative; PRMT, Partial remission under minimal therapy.

To find a prognostic marker for pemphigus after rituximab treatment, we analyzed B cells in PBMCs from 17 pemphigus vulgaris patients by flow cytometry using a gating strategy (**Supplementary Figure S1**). By using the recombinant human Dsg3 tetramer, we detected circulating Dsg3-specific B cells. There was no correlation between the time from the onset of pemphigus to rituximab infusion and the proportion of Dsg3-specific B cells among CD19^+^ B cells in patients (*p =* 0.314) (**Figure 3A**). We detected a few CD27^+^CD38^+^ antibody-secreting cells (ASCs), including CD24^-^CD38^+^ plasmablasts, among the circulating Dsg3-specific B cells. We subdivided non-naïve B cells into CD27^+^IgD^−^ switched memory B cells, CD27^+^IgD^+^ unswitched memory B cells, and CD27^−^IgD^−^ double-negative (DN) B cells. The relative frequencies of all three subtypes of non-naïve B cells in CD19^+^ B cells and Dsg3-specific B cells did not correlate with time to initiating rituximab (**Supplementary Figure S2**). However, among the three subtypes of memory B cells, we found that DN B cells were significantly enriched in Dsg3-specific B cells when compared to the total CD19^+^ B cells (**Figure 3B**). CD11c^+^ cells are enriched in Dsg3-specific DN B cells, and more than 90% of these cells are CD11c^+^FcRL5^+^ atypical memory B cells (**Figures 3C, D**). We thus investigated atypical memory B cells in Dsg3-specific DN B cells by using the CD11c marker. We found that the relative frequencies of CD11c^+^ atypical memory B cells in Dsg3-specific B cells were positively correlated with the time to initiating rituximab (R^2^ = 0.492, *p =* 0.002) (**Figure 3E**). Recognizing that the frequency of Dsg3-specific CD11c^+^ atypical memory B cells may have prognostic value, we conducted further analyses using other clinical outcomes. The frequency of this subset was positively correlated with the time from disease onset to PRMT (R^2^ = 0.485, *p* = 0.002) and the total amount of corticosteroid intake until the patient reached PRMT (R^2^ = 0.416, *p* = 0.005) (**Figures 3F, G**).

## Discussion

When evaluating the impact of the rituximab reimbursement policy of NHI, we found that the time to the first rituximab dose was shortened in the patients who were diagnosed after the reimbursement started. Thus, comparisons between groups before and after the reimbursement provide important evidence for understanding the effect of early rituximab treatment for pemphigus patients. In a previous study with 107 pemphigus patients treated with rituximab, the early treatment group (≤6 months after diagnosis) achieved a higher rate of remission than the late treatment group (>6 months after diagnosis) (15). In another study with 95 pemphigus patients receiving rituximab, the early treatment group achieved remission sooner and had a longer duration of remission (16). In addition to time to remission, we found a decrease in steroid intake before remission in the group diagnosed after the reimbursement, indicating that early-treated patients may experience a more rapid decline in the disease state after rituximab than late-treated patients.

ITS design is a powerful analysis for evaluating the longitudinal effects of a sudden change or intervention (17). Often combined with segmented regression, it visualizes the consequence of an intervention and clarifies its significance using a statistical approach (18). In the ITS analyses, we observed that the implementation of the reimbursement policy significantly reduced the trend of total steroid intake for the first year of treatment. Additionally, the amounts of steroids taken in the six months after rituximab infusion and until PRMT were both decreased in the patient group after the reimbursement. These results strongly suggest that early rituximab treatment is beneficial for patient outcomes. Contrary to the results from the ITS analysis, there was no difference in the total amount of steroids taken for six months after rituximab treatment when the two groups were directly compared directly. Given that ITS analysis reflects complex factors during treatment and incorporates underlying trends (19), we think that the findings from ITS analysis are more credible when comparing the trends before and after the intervention. Also, it is interesting to note that total steroid intake levels spiked at the time the rituximab reimbursement policy was initiated. We infer that many patients with severe pemphigus who could not afford rituximab were enrolled at that time. This real-world evidence highlights the value of ITS analysis.

Atypical memory B cells were first considered to be exhausted or activated B cells, but recent studies suggest that these cells are precursors of plasma cells mediated by the extrafollicular pathway (20, 21). They are observed in CD27^−^IgD^−^ DN B cells and are characterized by the overexpression of t-bet, FcRL5, and CD11c and downregulation of FcRL4, CXCR5, and CD21 (20, 22, 23). We also observed that most CD11c^+^ DN B cells were FcRL5^+^ cells in Dsg3-specific B cells from pemphigus vulgaris patients. An increase in atypical memory B cells is observed in various autoimmune diseases, including systemic lupus erythematosus, rheumatoid arthritis, Sjögren’s syndrome, and multiple sclerosis (24–27), and is associated with female dominance in autoimmunity (28). These cells have similar features as age-associated B cells, which are enriched in the old-age population (25, 29), and SARS-CoV-2-specific atypical memory B cells were increased in the peripheral blood of COVID-19 patients and vaccinees (13, 30).

Among Dsg-specific B cells, we suggest that Dsg-specific atypical memory B cells may forecast a favorable prognosis after rituximab treatment in pemphigus patients. Consistent with our data, a recent study showed an increase in CD11c^+^ B-cell frequency in pemphigus patients during active disease and a decrease after treatment (31). However, given that most autoreactive B cells in pemphigus undergo somatic hypermutation (5, 32), it is still unclear whether pemphigus is directly induced by Dsg-specific atypical memory B cells of the extrafollicular origin. Both pathogenic autoreactive ASCs in secondary lymphoid organs and autoreactive atypical memory B cells in peripheral blood can grow in number following disease progression. Since it is impractical to measure the frequency of pathogenic plasma cells in secondary lymphoid organs, we expect autoreactive atypical memory B cells to be a surrogate marker for predicting the prognosis of the disease and the response to rituximab.

After reimbursement policy, the period from the diagnosis of pemphigus to the initiation of rituximab has been significantly shortened, with substantial reductions in steroid doses throughout the course from the time of diagnosis. We also observed a positive correlation between the duration from the onset of the disease to rituximab infusion and the proportion of atypical CD11c^+^ B cells among Dsg3-specific B cells. Based on these findings, we expect that Dsg-specific ASCs will expand over time. The shorter the time to rituximab, the lower the number of Dsg-specific ASCs, so the disease can subside more quickly with lower doses of steroids. Although the time between the actual onset of the disease and rituximab infusion was not significantly different between the two groups, we believe that a further study with a larger cohort and prospective design may find a close relationship between the disease duration and the effectiveness of rituximab.

In summary, our study revealed that Korea’s reimbursement policy allowed early infusion of rituximab, which led to faster achievement of remission. A novel approach with ITS analyses provided evidence of a significant reduction in the trend of total steroid intake throughout the treatment period, which may relate to decreased morbidity and mortality. Furthermore, through PBMC analysis, we have highlighted the value of Dsg-specific CD11c^+^ atypical memory B cells as a prognostic marker for pemphigus patients.

## Data availability statement

The original contributions presented in the study are included in the article/**Supplementary Material**. Further inquiries can be directed to the corresponding authors.

## Ethics statement

The studies involving human participants were reviewed and approved by Gangnam Severance Hospital IRB No. 3-2021-0138 and No. 3-2019-0191. The patients/participants provided their written informed consent to participate in this study.

## Author contributions

The authors confirm contribution to the paper as follows: Study conception and design: AS, S-CK, JS, and JK. Data collection: AS and SL. Analysis and interpretation of results: JJ, AS, AL, SM, JK, and JS. Draft manuscript preparation: JJ, AS, and JK. All authors listed have made a substantial, direct, and intellectual contribution to the work and approved it for publication.

## Funding

This work was supported by the Amorepacific research fund, 2021.

## Conflict of interest

The authors declare that the research was conducted in the absence of any commercial or financial relationships that could be construed as a potential conflict of interest.

## Publisher’s note

All claims expressed in this article are solely those of the authors and do not necessarily represent those of their affiliated organizations, or those of the publisher, the editors and the reviewers. Any product that may be evaluated in this article, or claim that may be made by its manufacturer, is not guaranteed or endorsed by the publisher.

## Abbreviations

DN, double negative; Dsg, desmoglein; ITS, interrupted time series; NHI, National Health Insurance; PRMT, partial remission under minimal therapy; RTX, Rituximab.
