# Supplementary material for: Clinical impact and a prognostic marker of early rituximab treatment after rituximab reimbursement in Korean pemphigus patients
Source: Front Immunol. 2022 Aug 2;13:932909. doi: 10.3389/fimmu.2022.932909 (PMC9379325; doi:10.3389/fimmu.2022.932909)

Supplementary Material

**Supplementary Table S1.** List of dependent variables

| **Variable** | **Definition** |
| --- | --- |
| Time to first rituximab treatment | Time from the diagnosis of pemphigus to the first rituximab treatment |
| Total steroid intake over first year of treatment | Total steroid intake over the one year after the diagnosis of pemphigus |
| Total steroid intake over six months following rituximab treatment | Total steroid intake over the six months from the starting point of rituximab treatment |
| Time from onset to rituximab treatment | Time from the onset of pemphigus to rituximab treatment |
| Time to PRMT | Time from the diagnosis of pemphigus to the first PRMT |
| Total steroid intake after rituximab treatment until PRMT | Total steroid intake from the starting point of rituximab treatment to the first PRMT |
| Time from onset to PRMT | Time from the onset of pemphigus to PRMT |
| Total steroid intake to PRMT | Total steroid intake from the diagnosis of pemphigus to the first PRMT |
| Total steroid intake to CR | Total steroid intake from the diagnosis of pemphigus to the first CR |
| PRMT *Partial remission under minimal therapy*; CR *Complete remission* | |

**Supplementary Table S2. Comparison of rates of complete remission, relapse and complications between the patients diagnosed before rituximab reimbursement and after reimbursement^1^.**

| **Variables** | **Total** | **Before policy (2014.1. ~ 2018. 1.)** | | **After policy (2018.2. ~ 2020. 12.)** | | ***P* value^2^** |
| --- | --- | --- | --- | --- | --- | --- |
| **Complete remission** | 164 | 76 | (83.5) | 88 | (86.3) | 0.688 |
| **Relapse** | 47 | 22 | (25.0) | 25 | (26.9) | 0.866 |
| **Complications** | 16 | 10 | (9.5) | 6 | (5.5) | 0.306 |
| ^1^Data shown are number (percentage)  ^2^P values are calculated by the chi-squared test. | | | | | |  |

**Supplementary Figure S1. Gating strategy for flow-cytometric analysis of Dsg3-specific and CD19^+^ B cells of pemphigus vulgaris patients.**

**
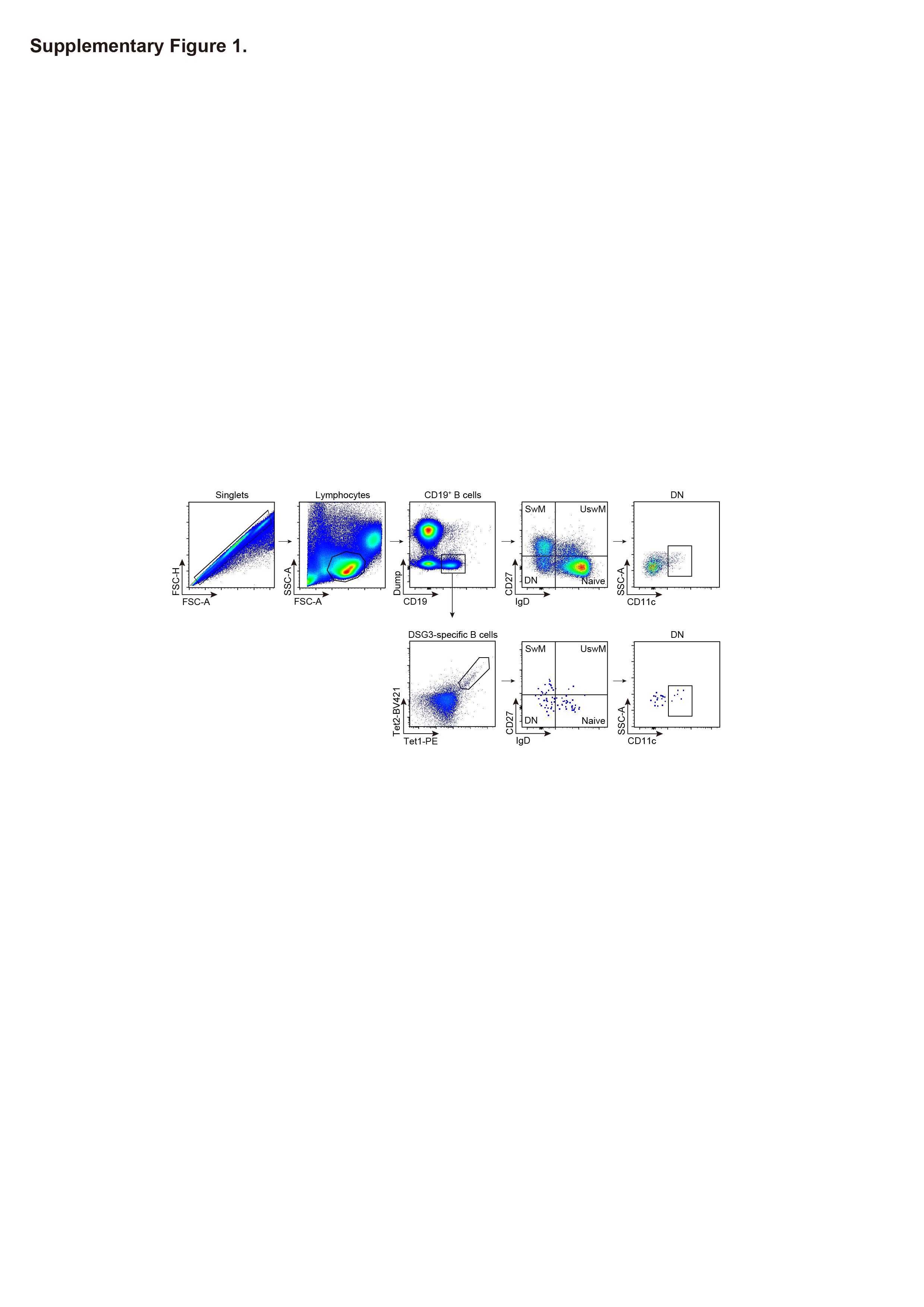
**

**Supplementary Figure S2. Correlation between the time from the onset of pemphigus to rituximab infusion and relative frequencies of B-cell subsets.** The three **s**catter plots on the first row show the correlations between the proportions of (A) switched memory (IgD^-^CD27^+^) B cells, (B) unswitched memory (IgD^+^CD27^+^) B cells and (C) DN (IgD^-^CD27^-^) B cells among CD19^-^ B cells and the time from the onset of pemphigus to rituximab treatment. The three in the second row display the correlations between the proportions of (D) switched memory B cells, (E) unswitched memory B cells and (G) DN B cells among Dsg3-specific B cells and the time from the onset of pemphigus to rituximab treatment.

Dsg *Desmoglein;* DN *Double negative; RTX Rituximab*


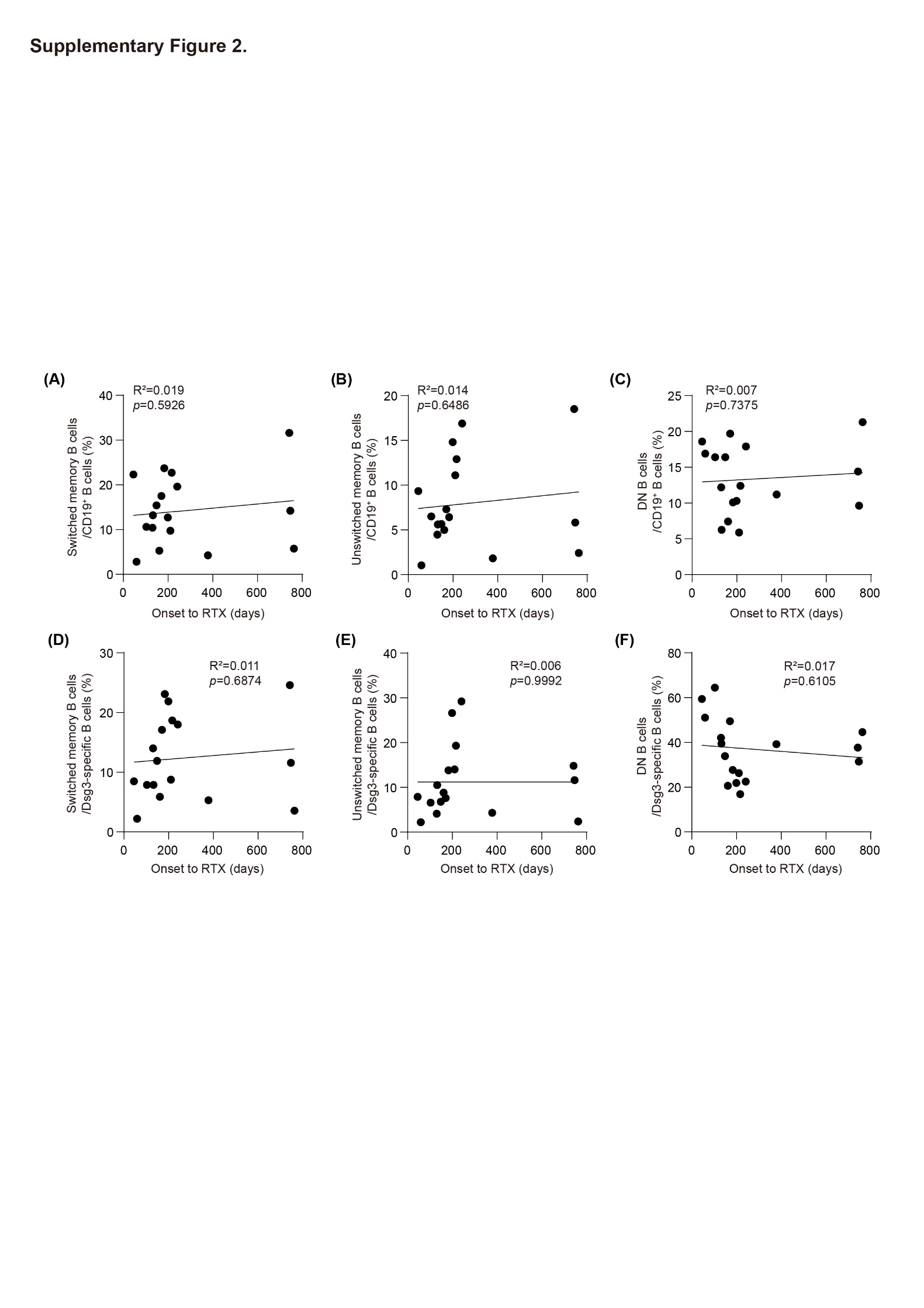

Supplement: Supplementary file 1 [file DataSheet_1.docx]
